# Learning Your Options: Option-Based Model of Export Readiness and Optimal Export

**DOI:** 10.3390/e24020173

**Published:** 2022-01-24

**Authors:** Kirill Ilinski

**Affiliations:** 1 National Economics Research Center (NERC), St.-Petersburg University, 7-9-11 Universitetskaya Embankment, 199034 St. Petersburg, Russia; kni@fusionam.com; 2 Fusion Group, 8-10 Great George St., London SW1P 3AE, UK

**Keywords:** export readiness, internationalization, options pricing

## Abstract

In this short note we offer a novel quantitative approach to modeling of early stages of firm’s internalization, namely stages of accumulation of export readiness and their export debut. In particular, we introduce a new model of export readiness and offer an explicit way of how the export readiness can be accounted in the company share price. The model considers export readiness as a non-observable intangible asset that changes a firm’s asset dynamics. This, in the framework of an option-based debt-equity Merton model, affects both the equity and debt of the company. The approach also allows one to define the contribution of export readiness to equity price and to find a self-consistent quantitative solution to the problem of optimal export strategy and the corresponding optimal firm’s capital allocation.

## 1. Foreword

This article is written specially for the issue of Entropy, to commemorate 30 years of Econophysics, the discipline which appeared at the beginning of the 1990s at the cross-roads of economics, mainly finance, and theoretical physics, in particular many-body systems, thermodynamics, and phase transitions. It was driven primarily by physicists who, being generally curious about nature but not particularly educated about the specific field at the time, did not see any barriers to tackle almost any problem in finance they could think of. These would be, for example, the non-liner modeling of market prices, derivatives pricing, and non-equilibrium market dynamics, to name a few. This intellectual effort, coupled with a cheeky belief in technical superiority and nearly barbaric economic ignorance led to the situation when, in a relatively short period of time, truly cross-disciplinary problems, previously overlooked or simply perceived, hopelessly too complex, were posed and tackled.

To keep up with the tradition and in the general spirit of things, we do not want to present here just another paper on portfolio theory, generalized Sharpe ratios, or a new market forecasting technique—all the boringly routine daily subjects for finance professionals, the things the author actually thinks he now knows about. Instead, we aim to sketch here a new quantitative solution to an old problem in a field that is relatively new to the author. We consider the problem of transition to internationalization through export, export readiness, and forecasting of export success–the questions lying in the overlap of several economic fields, namely Theory of the Firm, International Trade and Finance, and Economics of Government Intervention. The proposed model utilizes objects and methods familiar to us from the field of quantitative finance, in particular capital structure modeling and derivatives pricing, which to our knowledge have not been used in this context before. In this way, we try to keep up with the original Econophysics tradition of solving problems we knew very little about until recently, using methods that earn us our daily keep.

## 2. Long Introduction—Setting Up the Context

### 2.1. Internationalization

Accelerated globalization driven by political development, falling trade barriers, development in shipping, and advances in technology, has resulted in nearly 6-times growth in international trade since the 1970s. This growth is not just proportional to GDP growth—international trade accelerated quicker and increased from 10% to 25% of GDP, being one of the drivers of the world’s GDP increase.

Studies of how a firm undergoes internationalization—meaning how it expands sales from its own domestic market to some foreign markets, go back nearly 50 years and span a vast field of extensive research effort. We do not attempt here any sort of comprehensive review or introduction in the field and aim only to identify the main branches of the literature, especially in the context of the problem, which we address in this article. We will try to limit the list of citations in this short note to keep the balance but interested readers can find references in the articles for further reading. The most recent review of literature as well as another empirical study of link export readiness and export success can be found in the article [1].

Johanson and Vahlne in their seminal paper “The Internationalisation process of the firm: A model of knowledge development and increasing foreign commitments” (1977) [2] introduced what is now known as the original Uppsala model. The model views internationalization as a sequential process of firm development, from a pre-internationalization phase, to trial export, export through a partner, establishing a foreign subsidiary, and foreign manufacturing. In modern formulation, the model distinguishes a pre-export stage, “experimental” export with accumulation of experience, a committed exporter stage, and a full-integration (multi-national) stage. An updated Uppsala model (2009) [3] moved from individual interactions to interactions of economic agents inside a business network of contacts and a recursive learning process in this network. These are not the only model, of course—alternatives would include internationalization through business networks [4], “Born Globals” [5,6], model of “cultural distance” [7], among others. We, however, wanted to start with the Uppsala model because it clearly identified the pre-internationalization stage with its accumulation of key resources needed for a trial export as a battle ground to understand the process of crossing from non-exporting to an exporting firm.

The pre-internationalization stage can be viewed as a stage when a firm accumulates knowledge and resources to start an initial export [8]. The firm readies itself for export, i.e., it accumulates export readiness ([9] defines export readiness as preparedness and propensity to commence export). Multiple papers try to assess export readiness (as early as 1990, [10]) and to construct quantitative algorithms to estimate—find a number, an index—which would characterize export readiness of the firm, in hope that this number would define the corresponding success of future export.

It became clear quite early in the study that the firm’s readiness to export should be assessed from two angles: Operational readiness and product readiness [11]. This is reflected now in some modern two-stage export readiness tests, which first analyzes general organizational readiness and then overlays it with a “particular product for particular market” analysis [12,13]. Popular research directions in this area include studies of key factors for export readiness, construction of qualitative and quantitative questionnaires, and an evaluation of different methodologies of digitalization of particular qualitative characteristics/answers. All these become inputs into the construction of multiple-item indices to measure export readiness. The indices then tested through logistic regression in a large sample of companies to separate exporters from non-exporters. The Holy Grail of export readiness would be to find an export readiness model that is quantitatively built from both objective and subjective information about the company, such that it would be able to predict future out-of-sample success of the export activity, as well as being able to identify particular problem areas that need to be addressed to increase the chance of this success. It would also be good if the model can time the crossing to export, adding a time dimension to the problem, and explaining how the threshold is reached and when. Indeed, the firm can be “accumulating the knowledge” but never actually becomes involved in the export. How does this jump actually happen? Only an export readiness model with elements of the dynamics can fully and self-consistently answer the question. This article is a step in the quest for the Holy Grail.

### 2.2. Million Dollar Question—Export Readiness

The problem seems very academic and almost artificial. However, there are a couple of very practical angles to it.

#### 2.2.1. Government Support

First of all, governments, central and regional, try to support export. The importance of this is not purely economical but a socio-political one as well. Typically, it is done by creating specialized agencies whose main role is to educate the firms, promote international trade, and provide specific measures to stimulate firms to explore export sales. In Scotland, for example, the role is played by Scottish Development International and in South Africa it would fall to the Trade and Investment South Africa agency. Firms in Russia’s Moscow region (actually comparable to a not-so-small country) can find support from ANO Mosprom, a specialized government agency whose role is to increase the export of Moscow region enterprises. The support has its cost, paid by taxpayers money, which has to be spent in the most efficient way. One of ways to define the measure of this efficiency, one of possible Key Performance Indicators for the government agencies, in this case would be the amount of additional export generated by the firms per one dollar spent by the agencies. Adding to this, a limit of maximal annual aid per company and requirement on the minimal number of the supported firms per year, we come to problems regarding the efficient selection of candidates for export stimuli. The solution has to be objective, transparent, and sufficiently simple to be explainable because nothing is more damaging to budget spending than implications of cronyism and corruption.

Finding a quantitative solution is not a trivial problem because it requires prediction of future export success, which is particularly difficult for companies that have not entered the export market yet and are only planning to do so. Unfortunately, this is also the most practically interesting case for the agencies since the highest marginal effect of government support measures comes from these companies, which usually belong to the Small and Medium (SME) sector of the economy. There are literally millions of companies. One has to have a quantitative screening process to limit this number of hopefuls to a manageable quantity, so that a handful of “expensive” experts can look further. In short, to efficiently distribute government resources allocated to the support, we need to estimate how ready a non-exporting company is for export, what the corresponding probability of export success is, and what the monetary consequences of the company’s export would be. The latter question also requires an assumption about the internal optimal allocation of the firm’s resources to domestic and export markets, which in turn, again requires probabilistic assumptions about export success.

#### 2.2.2. Corporate Robo-Advice

Government agencies cannot help every company because of the associated costs involved in working with the candidates. Thus, they need to identify the candidates better (this is already covered above), but also to provide everybody else with a proxy service of export consulting, which does not require the same level of personal involvement from the agencies and can be done at virtually zero cost using online advice portals. This is what we call Corporate Robo-Advice, to underline the similarity with Financial Robo-Advice already widely available to individual retail customers whose assets are not sufficient to justify full face-to-face financial advice from specialized regulated financial advisory firms, or who are not prepared to pay full price for the advice. Instead, customers opt to receive proxy advice, generated online using risk profiling and asset allocation algorithms. Numerically, in developed economies, the numbers of potential clients of both Robo-advisories are actually quite similar and are sufficiently large. For example, in the UK number of people currently not receiving financial advice but wanting to receive it and one prepared to pay for it is close to 6 mln [14]. The number of active (employer) Small and Medium (SME) firms in the country is circa 1.4 mln, with the total number of private firms at about 5.5 mln [15]. The number of employer firms in the US would be close to 6 mln. Only the use of algorithms to provide tech-driven advice can help to close both “advice gaps”.

In the case of corporate clients, many agencies are keen to develop online portals that can efficiently estimate the export potential of firms. These portals qualitatively and quantitatively define firms’ export readiness and estimate the probability of export success, while at the same time provide concrete advice on improving areas of operations linked to all these quantities. Most export agencies, as well as some export-oriented banks (see for example, HSBC [16]), already have online resources with educational literature and simplified questionnaire-based models to estimate export readiness and, often, to highlight problem areas. These models are mostly qualitative, lack predictive power or statistical grounds, and play a primarily marketing role. The development of a full quantitative model of export readiness, forecasting of success probability, and the optimal export firm’s exposure is a necessary step in providing a quantitative advisory to SME firms in the context of export. This includes identifying the most important factors, statistically testable functional dependencies on these factors, particular firm’s shortfall in areas affecting these factors, and the most cost-efficient way to improve the outcome (so-called goal-oriented advice). This part of consulting should be an integral part of wider Corporate Robo-Advice including treasury advice, financial planning advice, budgeting, and hedging advice.

#### 2.2.3. Probability of Successful Export as Export Readiness Index

A historically popular approach to evaluating export readiness was a construction of numerical index (ex. [9,17]) based on the digitization of answers to a particular export readiness questionnaire and complementing this index with a threshold number—if an index for a particular company was above the threshold, the company was defined as export ready. The selection of questions was driven by a selection of factors that were assumed to be particularly relevant for the internalization initiation. The index is usually calibrated on a mixed set of exporting and non-exporting companies. Parametric ansatz for the probability of future export success can be seen therefore as a particular choice, probably most logical, of an export readiness index. The model described below for the increasing export-preparedness of a company and the corresponding dynamics of its export debut can be seen as an alternative approach to the construction of export-readiness indices, being structural rather than phenomenological.

## 3. Challenges in Formulating the Model

Hopefully, by now the reader is convinced that the problem at hand is an interesting one to study. So why has it not been already solved, if so much research effort and practical investment has been already dedicated to it? This is because it is notoriously complex, partially due to its ill-definition, multiple possible scenarios of internationalization as well as internal firm’s dynamics, and a multitude of factors affecting both the route and dynamics. On top of that, one of the most important factors in export readiness is the internal motivation of management and internal (read—cultural) specifics of the firm. A host of little issues can decide when, if at all, the firm decides to export. We argue here that in the context of multiple random or unknown factors, the dynamics of an export debut should be described by a stochastic process.

Before moving further, let us list common challenges which are facing every model of transition to export.

### 3.1. Challenge 1—What Is the Event?

Before we calculate a probability, we need first to define an event. We need to define the export success or a particular level of export efficiency which could be seen as a “success”. Is it a single, first export transaction? Is it a particular percentage of total revenues of a company coming from export activities? Is it reaching a particular level of export intensity (say, 10%, commonly taken as a border between “export experiment” and “active exporters”; or may be 40% to count the firm as a “committed exporter”)? Perhaps it is achieving a particular level of export efficiency? Or may be it is not quantitative at all and is defined by the perception of the firm’s management of satisfactorily achieving their export goals (which also may not be purely quantitative, such as reputation of an international firm, personal ambition, protection against political prosecution, etc.). This is not an idle question—all that we have just brought up as examples are, in fact, actual measures. Ref. [18] documents 45 measures of export efficiency. It is complemented by another paper [19], 4 years later, bringing the total listed number of different measures to 50. A comprehensive literature review [20] lists 9 main categories of determinants of export performance and 36 main export performance measures (referencing literally hundreds of scientific publications). The criteria to select a particular measure of export efficiency and, therefore, a definition of export success, is dictated by a wider context of the problem for which one has to find the probability (for example, specific target set of Key Performance Indicators, or a target function to be optimized for a particular agency’s development program). In practical terms, different definitions of export success will cause different calibrations of the same model on different information sets.

### 3.2. Challenge 2—What Is the Time Horizon?

We are looking for a probability of the event happening. Strictly speaking, this requires us to define a particular time window in which we observe firms to define whether the positive outcome has happened. What is this time window? Popular choices include 2 years and 5 years. Intuitively it seems that the dynamics of 2-year and 5-year windows are different, economically and functionally, and is led by potentially different factors. A reasonable model must describe this shift in relative importance of the factors, as well as potentially different functional dependencies on them. Basically, to have a self-consistent model for the export transition, we ideally need a model that would describe all windows, the whole term structure curve of probabilities of export success. Current Bayesian logit-linear models of construction of export readiness indices do not address this.

### 3.3. Challenge 3—Why Do Different Firms with the Same Parameters Behave So Differently?

Every firm is different—different corporate cultures, different styles of management, and a different speed of making corporate decisions (the corporate time). We can name so many various idiosyncratic factors that it is impossible, and also not actually desirable to account for them all. We are going to account for one of them—the corporate time, but will treat the rest in a reduced description approach, changing to a stochastic picture of internal firm’s dynamics and response to external macro stimuli. In this approach, similar initial conditions will define similar statistical behavior rather than exact matched outcomes. In short, we aim to build a stochastic model of the first (successful) export event and will calculate the probability of a successful export as a result of this model.

### 3.4. Challenge 4—What Is “Physical Meaning” of Export Readiness?

Firm needs to become “export ready” before considering physical investment into resources to access export markets. What is this “export readiness”? Increasing export-readiness, simply according to its definition, makes a company prepared to export successfully. Successful export changes the dynamics of assets of the company, adding new channel for assets growth, which comes with its own associated risks. Therefore, export readiness can be defined as a characteristic that becomes a signal variable for the change of the asset growth process. It is an intangible asset of the company which is, mostly, not reflected in the balance sheet of the company but is vital to defining company dynamics and valuation.

Thinking of intangibles in the context of change in the parameters, or even nature, of company growth is not a new concept. Intangible assets such as skilled workforce, patents and know-how, unique organizational design and processes, even corporate culture represent valuable investments. Export readiness can be seen as a particular type of intangible capital which is required in a necessary quantity to initiate export activity. This is the approach we take in this paper.

Intangible assets are generally divided as intangible capital and intangible effort. Intangible capital is the stock of capital a company possesses, while intangible effort is the expenses spent on developing and maintaining intangible capital. Different accounting treatment and, as a consequence, different tax treatment dictates a recorded split of intangible assets and, for our purposes, obscures the economic picture that could be tested. If the intangible assets are estimated from the split of associated costs analyzing financial information of a company, it is easier to test a positive relationship between an investment in intangibles and export intensity [21,22,23], but it ignores the fact that a lot of things cannot be priced and are not charged for. Management motivation would be one example. Therefore, here we opted not to consider export-readiness in the resource-based view and firm-specific asset theory [24] and model it within the assets of the firm. Instead,

**Proposition** **1.** *We see export-readiness as a stochastic variable that defines the asset process rather than a component of the assets.*

This is the main difference between our approach and the existing literature on the subject.

Thus, there is no standard way to measure a company’s intangible capital because there is no a single accepted definition of intangibles. There are many ways to measure it (paper [25] found nearly 700 papers related to measurements of intangible capital). In general, they are split into cost-based and value-based concepts. However, even in the cost-based approach, there is no single agreed method to define intangible expenses and no standardized accounting method to account for them in financial reporting. In simple terms—it is not clear what you need to add to the assets in the balance sheet, so that you can use structural model for the firm valuation, based on the same model for asset dynamics but with re-defined assets. Therefore, we here take a view that export-readiness, *R*, is a special type of intangibles for which we define a process which, in turn, will affect the dynamics of the standard (accounting) assets *A* of the firm. The value of the firm will then be calculated, as in the Merton structural model, as a price of a call option on the assets with the firm’s debt as a strike.

## 4. Quick Primer on the Structured Merton Model

Capital structure arbitrage models are a way to think about the relative pricing of debt and equity of a particular company. Everybody nowadays begin their introduction to the field with the Merton Model, for it is the simplest and most intuitive way to look at the matter. While the model, or rather the whole framework, is referred to as the Merton Model and his paper [26] is mostly cited in this regard, it is fair to add that Black and Sholes in their original paper [27] already considered corporate debt in the context of derivatives pricing. While there is an extensive body of literature on the capital structure models and various extensions and generalization of the Merton Model, we need here only a basic framework. Therefore, we will use its minimal set up, ignoring multiple complications and extensions (another 50 years of research).

Let us consider a company, ABC Limited. The company’s balance sheet will show the balance (the clue is in the word) of assets of the company and its liabilities, i.e., means of how these assets are funded. On the one hand side there, are assets, everything which ABC possesses. This might include machinery, stock, patents, leases, furniture, cash, etc. The total value of these assets at time *t* we denote as A(t).

On the other side, we have sources of capital that were used to finance these assets. These sources usually include debt *D* maturing, say, at time *T*, and the Book Equity Capital (Shareholder Funds). Book Equity balances the equation:

(1) A(t)=D+BookEquity(t)

at any moment of time and contains the initial equity investment from shareholders, subsequent equity placing proceeds, and importantly, the accumulated Profit and Loss of previous periods. As a little clue where it is all going, if assets do not contain some intangibles, like export readiness, Book Equity will not reflect this either. The Market Price of Equity, or offered share price, on the other hand will. Therefore, we need to go from Book Equity to Market Price of Equity.

Since Debt is maturing only at time *T* in future, its current value is not *D* but less, and it depends on the probability of the company being able to repay the debt. The insight of the original authors cited above was that both the current value of the debt and current value of equity do not coincide with Book (balance sheet) values but are both derivatives of the current asset value. Indeed, if at time *T* when Debt matures, the value A(T) is less than *D* then equity will be worthless and all assets will be sold to re-pay, as much as possible, the Debt:

$$D\left(T\right)=D-\left(D-A\left(T\right)\right)*\theta \left(D-A\left(T\right)\right)\equiv D-{\left(D-A\left(T\right)\right)}^{+}.$$

Here θ(.) is the Heaviside function. In option pricing, this “payout” corresponds to cash *D* and a short put option on the firm’s assets with strike *D* and maturity *T*.

At the same time, Equity would be equivalent to a call option:

$$E\left(T\right)=\left(A\left(T\right)-D\right)*\theta \left(A\left(T\right)-D\right)\equiv {\left(A\left(T\right)-D\right)}^{+}$$

with strike *D* and maturity *T*. To find values of both debt and equity for the company one has to use option pricing techniques which depend on the complexity of the asset dynamical process. If the process is a simple log-Brownian motion:

(2) $$dA=A\sigma_{A}dW_{A}$$

then one can quickly get simple analytical formulae for prices of both corporate debt and equity. In a more general case, the prices are values of the payout functions averaged with the transition probability of the asset values (assuming for simplicity zero interest rates):

$$E\left(t\right)=\int_{0}^{\infty }dA{\left(A-D\right)}^{+}*P\left(A\left(t\right),t,A,T\right)$$

$$D\left(t\right)=\int_{0}^{\infty }dA\left(D-{\left(D-A\right)}^{+}\right)*P\left(A\left(t\right),t,A,T\right)$$

where P(A(t),t,A,T)dA is the probability of the Asset value finishing in interval dA around *A* at time *T* conditional on the value of the asset being A(t) at time *t*.

These prices will still satisfy the balance condition of equity plus debt to be equal to the value of assets, which in option world is known as Call-Put Parity. If, for some reason, prices of equity and debt change so that the Call-Put Parity brakes, it causes a “risk-less” profitable (arbitrage) trading opportunity, exactly as it happens in option trading. This arbitrage is called Capital Structure Arbitrage to reflect that it is caused by dis-balance between different parts of the company’s capital structure.

Options that are used above are European vanilla options, meaning that their payouts are defined only by the value of the Asset at maturity. Black and Cox [28] removed this assumption by stating that for the company to avoid default, the barrier D should always remain un-breached, not only at maturity but also prior to maturity. This condition is called the American barrier, and it models an existence of loan covenants which, if breached, accelerate the debt repayments, thus bringing maturity forward. For our main purpose here, we will stick with the simplest Merton framework and will consider its basic European formulation, adding that all the usual refinements to the model (volatility skew, random barrier, complex debt profiles, etc.—see, for example [29]) can be added at a later stage.

## 5. Formulating the Model

### 5.1. Defining the Export Readiness Process

All studies of export-readiness first examine different factors affecting the export readiness. There are different taxonomies of the factors. The factors can be classified as intrinsic or external. They can be defined according to the mechanism of their action—for example, existing contacts with foreign partners, motivation of the management, sufficiency of financial resources, ability to manage risk, ability to modify its product, etc. These factors are typically selected by experts for a particular export-readiness model and are reflected in the corresponding questionnaire. Answers to the questions need to be digitized and the rule to combine the digital answers have to be defined. Examples of these workings can be found in [9,12,17]. The process is as much an art as it is a science and multiple trial-and-error iterations of the models have to appear before the model becomes operational. For our goals here we, however, will use a different classification—we will split factors between static (necessary to begin exporting) and dynamic (able to affect (increase or decrease) export readiness). For the combination of all static factors we will call export barriers, while the dynamic factors will be called export stimuli. Examples of components of export barriers would be: export licenses, knowledge of expected product support in export countries, ability to manage foreign exchange and interest rate risks etc. Examples of components of export stimuli would be company-sponsored foreign language lessons, government support, and education programs to increase awareness of foreign markets, management participation in industry networking events, etc. This classification does not remove the problem of building corresponding questionnaires and digitalization of qualitative answers but how it is done is not critically important for our subject here. It is enough for us to assume that all export barriers answers are digitized and combined in a total Export Barrier *B*. At the same time, the firm undertakes activities to increase/support/maintain export readiness while also fighting export readiness decay (example, people leave which reduces the expertise). The firm fights “Lateral rigidity” which (see [30]) is seen as one of the most important factors in export commencing. This results in change in export readiness per unit of time. The activity is reflected in the answers to the questionnaire. All Export Stimuli answers are digitized and combined, scaling for a unit of time, to obtain export readiness drift $\mu_{R}$. Even if the definition is somewhat arbitrary, it has to be consistent across all the companies to allow for effective model calibration. Now, let us assume that, according to a particular questionnaire, the firm is distance B far from the export barrier and has export drift $\mu_{R}$. The stochastic model for the export readiness *R* then takes the form: Initial *R*(0) = 0, export indicator ξ=0:

$$dR=\mu_{R}dt+\sigma_{R}dW_{R}$$

and the export event (ξ=1) is defined as *R* breaching the barrier B for the first time. Export-readiness volatility, the measure of uncertainty of the stochastic process, is a new parameter. This parameter characterizes measure of internal company dynamics—parameter $\frac{1}{\sigma^{2}}$ can be seen as a measure of internal company-specific time (different firms can have different speed of taking decisions, for example) as well as a measure of firm’s susceptibility to external noise. Export readiness *R* is therefore affected by random noise and by the drift which results from combined Export Stimuli.

In this formulation, the export readiness is an unobservable, hidden variable which reflects an increase of expertise and other resources required to begin export. It does, however, have two important derivative quantities that depend on it and can be estimated directly: Probability of export success and equity value of export readiness.

### 5.2. Probability of Export Success

The model allows analytical expression for probability of successful export on different time horizons, being simply the probability of breaching the barrier *B* for different time windows. From these probabilities we can build a curve of export exits that is similar to the CDS curve in credit derivatives. We can also explicitly calculate a probability distribution of time of successful export entry as a probability distribution of first passage time in the described above barrier problem. Explicit formulae for both quantities can be found in any textbook on probability theory. In particular, the probability distribution of the first passage time, the function which we will use below, for fixed barrier *B* and drift $\mu_{R}$ is given by the following expression [31,32]:

(3) $$\Pi \left(\tau \right)=\Pi_{\tau }=\frac{B}{\sigma_{R}\sqrt{2\pi }\tau^{3/2}}\exp\left[-\frac{{\left(B-\mu_{R}\tau \right)}^{2}}{2\sigma_{R}^{2}\tau }\right]$$

while the corresponding survival probability can be written as:

(4) $$\Pi_{T}=1-\int_{0}^{T}\Pi \left(\tau \right)d\tau =\Phi \left(\frac{B-\mu_{R}T}{\sigma_{R}\sqrt{T}}\right)-\exp\left(\frac{2B\mu_{R}}{\sigma_{R}^{2}}\right)\Phi \left(\frac{-B-\mu_{R}T}{\sigma_{R}\sqrt{T}}\right)$$

where Φ(.) is Cummulative Error Function. Both types of the quantities can be used to calibrate the model parameters to the existing information set of exporting companies, particular questionnaires, and particular selection of measurements of export success. In this form, the model is also able to explain the relative importance of different factors on different time horizons, since the effects of volatility dominate on shorter time horizons while the drift is the defining factor in the long run.

We end this sub-section with a note on the further use of the model framework rather than the simplified model for *R* itself. As in the Merton model for credit default, it was long argued that, while the hindsight of the model is definitely valuable, the log-Brownian asset dynamics are too restrictive. It forces us, through model calibration, to use “wrong parameters in the wrong model”. One of the approaches to estimate the probabilities of default was suggested by Vasicek and co-authors in the form of the KMV model [33], which, together with KMV Corporation, was acquired in 2002 and is included in services provided by Moody’s analytics. The main role in this approach was played by the distance to default which in the option picture corresponds to the moneyness. Using the analogy here we can introduce Distance to Export as:

$$DE=\frac{B-\mu T}{\sigma_{R}^{2}T}.$$

One can group companies according to the value of DE and plot probabilities of successful export as functions of DE. This functional form then substitutes of the Cumulative Error Function appearing in our simplified model and effectively corrects simplified the log-Brownian dynamic assumption. The model can be further expanded for the practical use applying the same technique as in the KMV model in the context of Export Readiness and substituting Distance to Default with Distance to Export.

### 5.3. Equity Value of Export Readiness

The model allows one to find “observable” equity value of un-observable export readiness. To this end we are to use the Merton model and see how the price of equity changes due to a possible change of asset dynamics if there is a possibility of a new export channel.

We saw above that equity price E of the company can be calculated as a price of call option on the assets *A* of the company. However, now, rather than to follow asset process (2) assets *A* of the company, ABC Limited will follow a modified Merton stochastic process with a switch from pure domestic to domestic+export dynamics triggered by export readiness variable R reaching the export barrier *B*.

#### Simplified Toy Model

In our toy model, we substitute a simplified assumption for the asset process (2):

$$dA=A\sigma_{A}dW_{A}$$

with a more complicated asset process:

$$dA=A\mu_{A}dt+A\sigma_{A}dW_{A}$$

where the parameters are defined as:

$$\mu_{A}=\mu_{0}\left(1-\xi \right)+\xi \mu_{1},$$

$$\sigma_{A}=\sigma_{0}\left(1-\xi \right)+\xi \sigma_{1}.$$

Here $\mu_{0}$ and $\sigma_{0}$ correspond to the company’s evolution in a pure domestic market, and $\mu_{1}$ and $\sigma_{1}$ correspond to evolution of the assets of the company if both domestic and export channels are used. The variable ξ={0,1} is the same signal variable already introduced in Section 5.1 in the context of dynamics of export readiness *R*. We also assume that processes $W_{A}$ and $W_{R}$ are independent, in particular that:

$$<dW_{A},dW_{R}>=0.$$

In this case, equity price can be calculated as:

$$E{\left(t\right)}_{\mu_{R},B}=\int_{0}^{\infty }dA{\left(A-D\right)}^{+}*P_{R}\left(A\left(t\right),t,A,T\right)$$

where the transition probability $P_{R}\left(A\left(t\right),t,A,T\right)$ accounts now also for the switch to export. Introducing τ as a first passage time (to export barrier B) one can see that $P_{R}\left(A\left(t\right),t,A,T\right)$ can be calculated as:

$$P_{R}\left(A\left(t\right),t,A,T\right)=\Pi_{T}P_{0}\left(A\left(t\right),t,A,T\right)+\int_{0}^{T}d\tau \Pi_{\tau }*{\tilde{P}}_{0}\left(A\left(t\right),t,A,T\right)$$

where $\Pi_{T}$ is the probability of not touching the barrier from time *t* to time *T* (survival probability (4) with *T* substituted by T−t), $\Pi_{\tau }$ is the probability of first passage time being τ ((3) with *T* substituted by T−t), $P_{0}\left(A\left(t\right),t,A,T\right)$ is the log-normal transition probability distribution of $\frac{A}{A(t)}$ with parameters $\mu_{0}$ and $\sigma_{0}$ and, finally, ${\tilde{P}}_{0}\left(A\left(t\right),t,A,T\right)$ is the log-normal transition probability distribution of $\frac{A}{A(t)}$ with parameters $\tilde{\mu }$ and $\tilde{\sigma }$:

$$\tilde{\mu }=\frac{\tau }{T}\mu_{0}+\frac{T-\tau }{T}\mu_{1},$$

$${\tilde{\sigma }}^{2}=\frac{\tau }{T}\sigma_{0}^{2}+\frac{T-\tau }{T}\sigma_{1}^{2}.$$

These formulae give a semi-analytical solution for the equity price in the case of possible future exports. They also allow us to define the export readiness benefit to the shareholders, which is not reflected in the balance sheet of the company. The quantity, which we call the Export Readiness Benefit (ERB):

$$ERB=E{\left(t\right)}_{\mu_{R},B}-E{\left(t\right)}_{\mu_{R},B=\infty }$$

defines the monetary contribution of non-observable export readiness into the price of company equity.

### 5.4. Extended Model

A more realistic but unfortunately more complicated model can be built if we explicitly describe uncertainty in the domestic and in the export channels. In this case the asset process will take the form:

$$dA=A(\left(1-\xi \right)\left[\mu_{d}dt+\sigma_{A,d}dW_{A,d}\right]+$$

$$\xi [\left(\omega_{d}\left(t,A\right)\mu_{d}+\omega_{e}\left(t,A\right)\mu_{e}\right)dt+\omega_{d}\left(t,A\right)\sigma_{d}dW_{A,d}+\omega_{e}\left(t,A\right)\sigma_{e}dW_{A,e}]).$$

Here we introduced two sets of parameters, with subscripts *i* and *e*, which correspond to domestic and export markets, together with a new element—weightings of capital allocations towards the domestic and export markets, $\omega_{d}$ and $\omega_{e}$. Two Brownian motions, $W_{A,d}$ and $W_{A,e}$ describe the corresponding uncertainties in return from domestic and export markets. In general setup, all three Brownian motions, $W_{A,d}$, $W_{A,e}$, and $W_{R}$ are mutually correlated. The complexity of the model is not only due to the increased dimensionality of the problem. It is also due to the dynamical nature of changes in the optimal capital allocation between foreign and domestic markets. The weights $\omega_{d}$ and $\omega_{e}$ have to be found self-consistently from the problem of optimization of a particularly selected utility function from the firm’s equity value. This is a highly non-linear stochastic problem.

### 5.5. Self-Consistent Model for Optimal Export Strategy

This is still not the end of the whole story yet. The model for export-readiness process:

$$dR=\mu_{R}dt+\sigma_{R}dW_{R}$$

contains parameters that we have so far held constant. The company can decide to invest more (or less) into export readiness, spending some of the cash accounted in assets (thus adding negative drift into the asset process to account for spent cash) for change in the parameters of export readiness process—bringing more qualified staff, engaging a consulting company, and so forth. Luckily, the problem of the first passage time with time-dependent parameters has been solved by physicists [34] and some explicit formulae exist instead of the simplest expressions (3) and (4), bringing back physicists into the picture. Exchanging cash for change in the values of export barrier *B* and export stimuli $\mu_{R}$ is a management decision. This decision, once again, is driven by the same utility optimization problem. This makes the optimization problem even more complicated but now complete. The solution of the problem, which would give optimal spending on export readiness as well as optimal capital allocation weights, constitutes a self-consistent solution of an optimal export problem.

Let us pose the problem more formally. One has to choose the control functions to maximize the sharefolder value:

$$max_{\left(\omega_{d}\left(.,.\right),\omega_{d}\left(.,.\right),f\left(.,.\right)\right)}E_{T}\left(A_{0},B_{0},\mu_{R,0},\sigma_{R,0}\right)$$

where

$$E\left(t\right)=\int_{0}^{\infty }dA{\left(A-D\right)}^{+}*P_{R}\left(A\left(0\right),0,A,T\right)$$

and $P_{R}\left(A\left(0\right),0,A,T\right)$ is the transition probability for the asset process with the explicit “cash drain” term f(t):

$$dA=A(\left(1-\xi \right)\left[\mu_{d}dt+\sigma_{A,d}dW_{A,d}-f\left(t,A\right)dt\right]+$$

$$\xi [\left(\omega_{d}\left(t,A\right)\mu_{d}+\omega_{e}\left(t,A\right)\mu_{e}\right)dt+\omega_{d}\left(t,A\right)\sigma_{d}dW_{A,d}+\omega_{e}\left(t,A\right)\sigma_{e}dW_{A,e}]).$$

The signal variable ξ is defined, as before, by the export readiness process: Initial ξ(0)=0 and becomes ξ(τ)=1 when the export readiness process R(τ)(R(0)=0):

$$dR=\mu_{R}\left(t\right)dt+\sigma_{R}\left(t\right)dW_{R}$$

is breaching the barrier B(τ) for the first time at the first passage time τ. Time-dependent parameters of the export readiness process then are functions of the “cash drain”, which we take for simplicity to be linear:

$$d\mu_{R}\left(t\right)=m*f\left(t,A\right)Adt\;,\;d\sigma_{R}\left(t\right)=s*f\left(t,A\right)Adt\;,\;dB_{R}\left(t\right)=b*f\left(t,A\right)Adt\;$$

with some company-specific efficiencies constants *m*,*s*,*b*.

The solution to the combined problem is not “one fits all” as internal company specifics, the internal cost of changing export readiness parameters, and internal return profiles from domestic and export activity depends on a particular company. Solving this problem opens the way to a quantitative selection criteria for government agency early export support, which we highlighted in the Introduction.

## 6. Conclusions

In this short note we sketched a new approach to modeling export readiness dynamics and posed the problem of finding an optimal firm’s strategy of export debut. While the model is quite complex and requires a combination of analytical and numerical studies, it builds a qualitative and intuitive picture of the transition to export dynamics. The most labor-intensive component of further work is to construct a qualitative questionnaire and the corresponding quantitative digitalization algorithms to estimate the model parameters Export Barrier *B*, Export Stimuli $\mu_{R}$, and internal volatility $\sigma_{R}$ for different types of companies. As the size of the company can be one of quantitative factors affecting export readiness, it is possible that *B*, $\mu_{R}$, and $\sigma_{R}$ will be asset-dependent, which will further increase the non-linearity of the problem, causing multiple equilibria and conditional instability typical for this type of complex systems, bringing it even closer to problems studied by Econophysics. 

## Data Availability

Not applicable.
